# Exercise-Induced Modulation of Angiotensin II Responses in Femoral Veins From 2-Kidney-1-Clip Hypertensive Rats

**DOI:** 10.3389/fphys.2021.620438

**Published:** 2021-04-07

**Authors:** Agnaldo Bruno Chies, Maria Angélica Spadella, Priscila Ramos de Oliveira, Raquel Fantin Domeniconi, Talita de Mello Santos, Roseli Peres Moreira, Carla B. Rosales, Dulce Elena Casarini, Luis Gabriel Navar

**Affiliations:** ^1^Laboratory of Pharmacology, Marília Medical School, Marília, Brazil; ^2^Human Embryology Laboratory, Marília Medical School, Marília, Brazil; ^3^Department of Anatomy, Institute of Biociences, UNESP, Botucatu, Brazil; ^4^Department of Medicine, Nephrology Division, Escola Paulista de Medicina, Universidade Federal de São Paulo, São Paulo, Brazil; ^5^Department of Physiology and Hypertension and Renal Center of Excellence, Tulane University School of Medicine, New Orleans, LA, United States

**Keywords:** angiotensin II, endothelin-1, exercise, femoral vein, 2K1C hypertension, nitric oxide, prostanoids

## Abstract

The present study investigated the angiotensin II (Ang II) responses in rat femoral veins taken from 2-kidney-1clip (2K1C) hypertensive rats at 4 weeks after clipping, as well as the effects of exercise on these responses. In this manner, femoral veins taken from 2K1C rats kept at rest or exposed to acute exercise or to exercise training were challenged with Ang II or endothelin-1 (ET-1) in organ bath. Simultaneously, the presence of cyclooxygenase-1 (COX-1) and cyclooxygenase-2 (COX-2) were determined in these preparations by western blotting. In these experiments, femoral veins exhibited subdued Ang II responses. However, after nitric oxide (NO) synthesis blockade, the responses were higher in the femoral veins taken from animals kept at rest [0.137(0.049–0.245); *n* = 10] than those obtained in trained animals kept at rest [0.008(0.001–0.041); *n* = 10] or studied after a single bout of exercise [0.001(0.001–0.054); *n* = 11]. In preparations in which, in addition to NO synthesis, both the local production of prostanoids and the action of ET-1 on type A (ET_A_) or B (ET_B_) receptors were inhibited, the differences induced by exercise were no longer observed. In addition, neither ET-1 responses nor the presence of COX-1 and COX-2 in these preparations were modified by the employed exercise protocols. In conclusion, NO maintains Ang II responses reduced in femoral veins of 2K1C animals at rest. However, vasodilator prostanoids as well as other relaxing mechanisms, activated by ET_B_ stimulation, are mobilized by exercise to cooperate with NO in order to maintain controlled Ang II responses in femoral veins.

## Introduction

During exercise, there is a significant increase in metabolic demand, especially in cardiac, and working skeletal muscle (Delp and O’Leary, 2004; Duncker and Bache, 2008). To meet this metabolic demand an extensive blood flow redistribution occurs characterized by increases in blood flow to the heart, diaphragm and working skeletal muscles at the expense of flow to skin, stomach, intestines, pancreas, liver, kidneys, spleen, and non-working skeletal muscles (Armstrong et al., 1987; Musch et al.,1987a,b). It has been proposed that the exercise-induced blood flow redistribution may be facilitated by locally produced substances that modulate the vasomotor effects of angiotensin II (Ang II) in several vascular beds (Kashimura et al., 1995; Chies et al., 2010, 2013, 2017; Park et al., 2012). These studies suggest that local substances are released as a consequence of exercise-induced shear stress.

The effects of exercise upon the modulating mechanisms that regulate the vascular responses to endogenous vasoconstrictors are more clearly delineated in the arterial than in the venous beds. This is an important knowledge gap considering that, at rest, about 60–80% of the blood in mammals is localized in the venous compartment and a significant part of this volume may be shifted to the heart and muscle tissue during exercise (Rothe, 1983; Pang, 2001). Thus, an uncontrolled venoconstriction could result in an increment of resistance to centripetal blood flow, thus impairing the venous return (Rowell, 1993).

Exercise training promotes functional adaptations in rat portal vein that result in stronger Ang II responses whenever the animal is re-exposed to the exercise (Chies et al., 2010). Such greater Ang II responses apparently involve a balance between nitric oxide (NO) and vasodilator prostanoids, prevailing at rest, and endothelin-1 (ET-1) that prevails when the animal is subjected to exercise. Inhibition of NO synthesis increases the Ang II responses in femoral veins taken from sedentary animals kept at rest, but does not change such responses in preparations taken from animals submitted to a single bout of exercise or in those kept in training for 8 weeks. Apparently, mechanisms related to vasodilator prostanoids and/or some other vasodilator mechanism triggered by the action of ET-1 on ET_B_ receptors (ET_B_) act as backup to NO in animals exposed to exercise, and thus maintain control of the Ang II responses in femoral veins (Chies et al., 2013).

Considering that the local modulating mechanisms described are necessary to keep the Ang II responses in femoral veins under control during exercise, it is important to assess how they are altered in pathophysiological conditions that involve cardiocirculatory modifications and endothelial dysfunction. The 2-kidney-1-clip (2K1C) rat model develops, in parallel to the blood pressure elevation, an oxidative stress-induced endothelial dysfunction (Ceron et al., 2010; Xue et al., 2015; Kumral et al., 2016). This experimental model is particularly interesting during the phase I (up to 4 weeks) because it is also characterized by elevations in the plasma and tissue Ang II levels (Martinez-Maldonado, 1991; Nakada et al., 1996; Xie et al., 2006). However, high levels of Ang II have not been consistently reported (Guan et al., 1992). Accordingly, the aim of the present study was to establish the effects of a single short bout of exercise as well as of regular exercise training on the Ang II responses in femoral veins taken from 2K1C rats.

## Materials and Methods

### Animals

Eighty-six Male Wistar rats (350–450 *g*) were housed in plastic cages (50 cm × 40 cm × 20 cm) with four animals per cage. Food and water were available *ad libitum*. During the exercise protocol, rats were maintained in the training room under a 12 h light-dark cycle, with lights on at 07:00 h. Room temperature was maintained at 25°C. All procedures were performed in accordance with ethical principles (Guide for the Care and Use of Laboratory Animals, 1996), and the study was reviewed and approved by the Research Ethics Committee of the Marília Medical School (Protocol no. 300/14).

### Distribution of Animals Among the Experimental Groups

Animals were distributed to the experimental groups, taking into account their innate exercise capacity (Melo et al., 2003). 2-month-old rats were trained by daily 10 min treadmill sessions, for 2 weeks (Movement Technology LX 170) without inclination, at speeds of 0.3 to 0.5 km/h. At the end of this period, these animals were submitted to the maximal exercise capacity test on treadmill. In this test, the animals were placed on a treadmill at a speed of 0.3 km/h. Then, every 3 min, the belt speed was increased by 0.3 km/h until exhaustion. The time that the animals continued to run under these conditions reflects their “maximal exercise capacity.” Based on this parameter, the animals were distributed among the groups so that all groups received animals that, on average, presented similar results in the exercise capacity test. The following experimental groups were constituted: RS = resting-sedentary, consisting of sedentary animals studied at rest; ES = exercised-sedentary, consisting of sedentary animals submitted to a 20 min session of exercise with speed of 60% of the average maximum velocity (equivalent to the first session of exercise training) immediately before the organ bath experiments; RT = resting-trained, consisting of trained animals studied at rest; and ET = exercised-trained, consisting of trained animals studied immediately after the last session of exercise, respectively. Based on the maximal exercise capacity test, we also determined the average speed of each group.

### Exercise Training Protocol

The animals designated as “trained” (belonging to RT and ET groups) were exercised 5 days per week for 1 h per day for 8 weeks. The exercise intensity was progressively increased by a combination of time and velocity, attaining 1 h per day at a velocity corresponding to 60% of average speed of each group by the third week. This protocol has been defined as constituting low-intensity exercise training (Melo et al., 2003; Martins et al., 2005).

### Hypertension 2K1C

Rats were anesthetized with tribromoethanol (250 mg/kg, i.p) and subjected to a midline laparotomy to access the left renal artery. A silver clip (0.25 mm) gap was placed on the renal artery. After surgery, animals received a dose of enrofloxacin (Baytril^®^; 25 mg/kg^–1^, i.m) and dipyrone (300 mg/kg^–1^; i.m.). Another dose of dipyrone was given 12 h after surgery. The systolic blood pressures of these animals were measured before, as well as 3 weeks after the clipping surgery using the “tail cuff” method. Hypertensive rats presented systolic pressures greater than 150 mmHg and elevation of blood pressure ≥30 mmHg in comparison to the pre-clipping condition.

The clipping surgery occurred during the exercise training period (4 weeks prior to euthanasia for vascular reactivity experiments). Because the vasoconstriction induced by Ang II is the focus of the present study, the femoral veins were harvested during this time, which corresponds to the end of the acute phase I. This phase is known as the renin-dependent phase and is characterized by elevation in the plasma and tissue Ang II levels (Martinez-Maldonado, 1991; Xie et al., 2006). After this surgery, the rats were not exercised for 4 days, so that they could recover.

For plasma Ang II quantification, some RS normotensive (two kidneys; 2K) animals were used. These animals were submitted to the laparotomy, but without placing the clip on the renal artery.

### Plasma Ang II Level Quantification

Animals were killed in a CO_2_ chamber and blood samples were immediately harvested by caval puncturing with a syringe containing ethylenediaminetetraacetic acid (EDTA). The blood samples were centrifuged at 4°C (3,500 rpm) in order to separate the plasma, followed by addition of a mix of protease inhibitors (cOmplete mini; Roche). The samples were immediately frozen at −80°C for later quantification of Ang II.

The extraction of Ang II was held in Oasis HBL 3cc columns (Waters, Ireland), previously activated with methanol (5 mL), tetrahydrofuran (5 mL), hexane (5 mL), methanol (5 mL), and water (10 mL). After sample introduction, columns were washed with water (10 mL) and peptides of interest were eluted with ethanol, acetic acid, and water (90: 4: 6). The eluted fractions were lyophilized and ressuspended in 500 μL mobile phase A: 5% ACN (50 mL) in 0.1% orthophosphoric acid (1 mL), and filtered with 0.22 μm membrane before analysis. The Ang II was quantified by ELISA, using the “Angiotensin II Enzyme Immunoassay Kit” (SPI-DIO), according to the manufacturer’s instructions.

### Organ Bath Studies

Immediately after euthanasia and blood sample collection, the femoral veins were harvested, dissected, and divided into rings (3–4 mm) and set up in 2 mL organ baths. In the organ bath, the preparation was placed between two stainless-steel hooks, one of them bound to a stationary support and the other, connected to an isometric force transducer. Preparations were bathed in Krebs–Henseleit solution (composition in mmol/L): NaCl 130; KCl 4.7; CaCl_2_ 1.6; KH_2_PO_4_ 1.2; MgSO_4_ 1.2; NaHCO_3_ 15; glucose 11.1. The solution was kept at pH 7.4 and 37°C and bubbled continuously with a mixture of 95% O_2_ and 5% CO_2_. Tension was monitored continuously and recorded using a Powerlab 8/30 data-acquisition system (ADInstruments, Castle Hill, NSW, Australia). Prior to administering drugs, rings were equilibrated for 60 min at a resting tension of 0.5 *g*. The time frame from the end of the exercise sessions to the beginning of the Ang II cumulative concentration–response curves was approximately 90 min.

The responses (g) evoked by cumulatively adding Ang II (10^–11^ to 10^–7^ mol/L; Sigma) directly into the organ bath were plotted in Prism^®^ software to obtain concentration–response curves. From these, the area under curve (AUC) was calculated. The AUC, instead pD2 (negative logarithm of the concentration that evoke 50% of the maximal response), was used because not all curves showed a sigmoidal profile. Some preparations challenged with (ET-1; 10^–11^ to 10^–6^ mol/L; Sigma) showed a sigmoidal profile, thereby permitting to obtain the pD2. The R_max_ (maximum response), which is the highest response evoked by Ang II or ET-1 in each preparation was also determined.

The actions of Ang II were also evaluated in preparations pretreated for 20 min with 10^–4^ mol/L L-NAME and 10^–5^ mol/L indomethacin, non-selective NO synthase and cyclooxygenase inhibitors (Sigma), respectively, 10^–6^ mol/L BQ-123 (antagonist of endothelin receptor type A – ET_A_; Sigma) or 10^–6^ mol/L BQ-788 (antagonist of ET_B_; Sigma), 10^–4^ mol/L tiron (superoxide anion scavenger; Sigma), and 10^–4^ mol/L apocynin [a non-selective NAD(P)Hoxidase inhibitor; Sigma]. Some preparations were also studied in presence of PD 123,319, a selective inhibitor of angiotensin receptors subtype 2 (AT_2_). In parallel, the actions of ET-1 were evaluated in presence of both 10^–4^ mol/L L-NAME and 10^–5^ mol/L indomethacin. All drugs were administered directly into the organ bath.

### Western Blotting

Samples of femoral veins were collected, frozen in liquid nitrogen and stored at −80°C to be analyzed by Western blotting. Frozen samples were homogenized at 4°C in RIPA buffer (BioRad^®^, United States) with protease inhibitor (Sigma-Aldrich^®^, United States) in Tureaux type homogenizer for three 5-s cycles. The homogenate material was centrifuged at 15,000 rpm for 20 min at 4°C and the supernatant was collected. The protein quantification was performed as described by Bradford (1976) in ELISA plates with 96 wells, and read in an ELISA reader (595 nm). Aliquots (30 μg protein) were treated with buffer solution to run gel (Laemli Sample Buffer – BioRad^®^) and β-mercaptoethanol at 95°C for 5 min. The proteins were then separated by vertical electrophoresis (Mini-Protean, BioRad^®^) 7.5% polyacrylamide gel SDS-PAGE, and after the electrophoresis, transferred to a nitrocellulose membrane in a wet transfer system. Nonspecific protein binding was blocked with 3% skimmed milk in TBST buffer for 1 h at room temperature. The membranes were then incubated overnight with primary antibodies: COX-1 (4841; concentration 1:500; Anti-Rabbit, Cell Signaling Technology^®^, Danvers, MA, United States) and COX-2 (ab15191, concentration 1:500,Anti-Rabbit, Abcam Inc.^®^, Cambridge, MA, United States) all of which were diluted in 1% BSA in TBST. Subsequently, the membranes were washed in TBST for three 10-min cycles, incubated for 2 h with specific HRP secondary antibody (clone: ab97051, IgG goat-Anti-Rabbit concentration 1:1000, Abcam Inc.^®^, Cambridge, MA, United States; clone: a5420) diluted in 1% BSA in TBST, and then washed in TBST for three 10-min cycles. Immunoreactive components were revealed using a luminescence kit (Amersham^TM^ ELC Select^TM^ Western Blotting Detection Reagent, GE Healthcare^®^, United Kingdom) and the optical density of each band was measured using Image J^®^ Windows^®^ software, normalized by GAPDH (G3PD, concentration 1:1000, Sigma-Aldrich Co.^®^, St. Louis, Mo, United States) density.

### Statistical Analysis

The data normality was verified by the Kolmogorov–Smirnov test with Lilliefors correction and the homogeneity of the variances by the Levene test. The variables with parametric distribution are presented as mean ± standard error of the mean (±S.E.M.) whereas the variables without parametric distribution are presented as median and interquartile range (25th first quartile; 75th third quartile). When the parametric distribution of the data was verified, Anova-one-way test, followed by the Bonferroni *Post-Hoc* test, was used for comparisons among independent groups. Blood pressure values were compared by mixed Anova of repeated measures. When the non-parametric distribution of the data was verified, Kruskal–Wallis test, followed by the peer-to-peer comparison by the Mann–Whitney test with the Holm-Sidak *Post-Hoc* correction, was used for comparisons among independent groups. The significance level adopted was 5% (*p*-value ≤ 0.05) and the data were analyzed using SPSS software (version 19.0).

It is noteworthy that Rmax and AUC did not present a normal distribution and/or the homogeneity of the variances, therefore, they were analyzed by a non-parametric test and presented in the tables as median and interquartile range. However, the concentration-response curves, from which these parameters were calculated, were built by mean ± S.E.M. in order to facilitate the visualization.

## Results

### Systolic Blood Pressure and Plasma Ang II Levels

**FIGURE 1:**
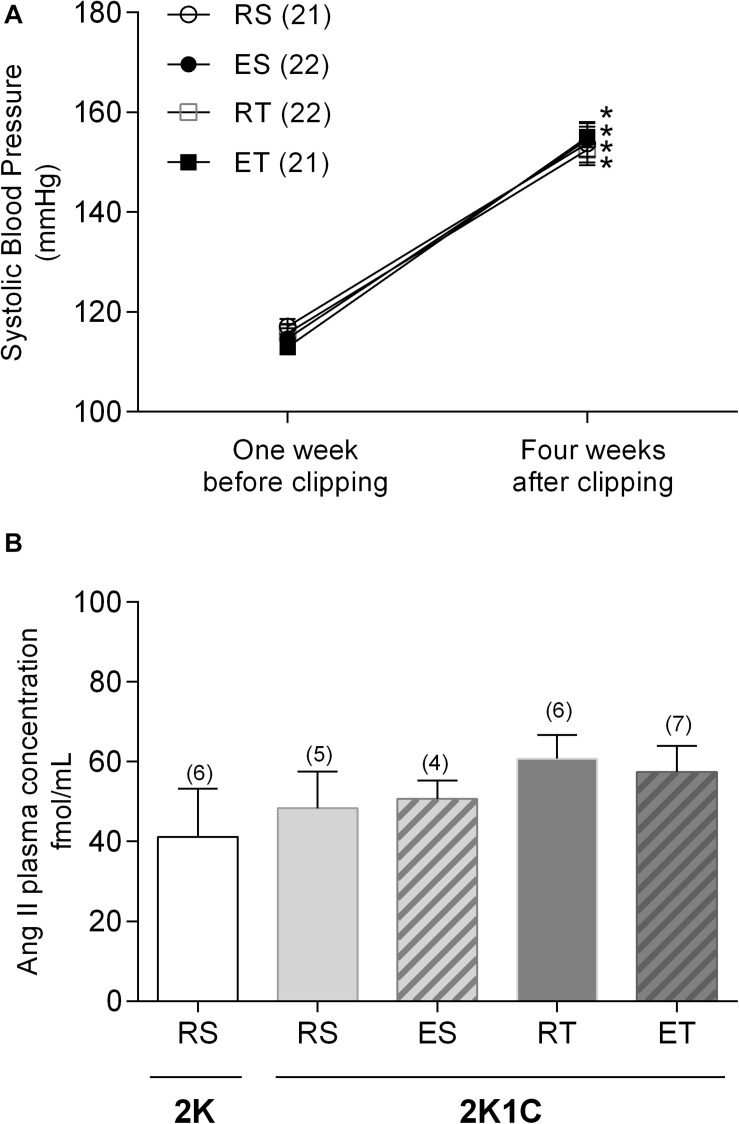
Systolic blood pressure **(A)** and plasma angiotensin II concentration **(B)** determined in 2K1C rats belonging to resting-sedentary (RS), exercised-sedentary (ES), resting-trained (RT), and exercised-trained (ET) groups. **(B)** also show plasma Ang II concentrations determined in resting-sedentary 2K animals. Values expressed as mean ± SEM; the number of independent determinations is in parentheses. *indicates significant difference (*p* < 0.001) from baseline in the same group (repeated measures ANOVA, followed by Bonferroni post-test).

The clip placement around the left renal artery led to a significant increase of systolic blood pressure in all studied groups (Figure 1A). These animals, designated as 2K1C, were demonstrably hypertensive in the day of the organ bath experiments. However, there was no difference in plasma Ang II concentrations between 2K1C and 2K animals when they were not submitted to exercise. In addition, differences in plasma Ang II levels were also not observed between 2K1C rats kept at rest and those submitted to any kind of exercise (Figure 1B).

### Vascular Responsiveness

**FIGURE 2:**
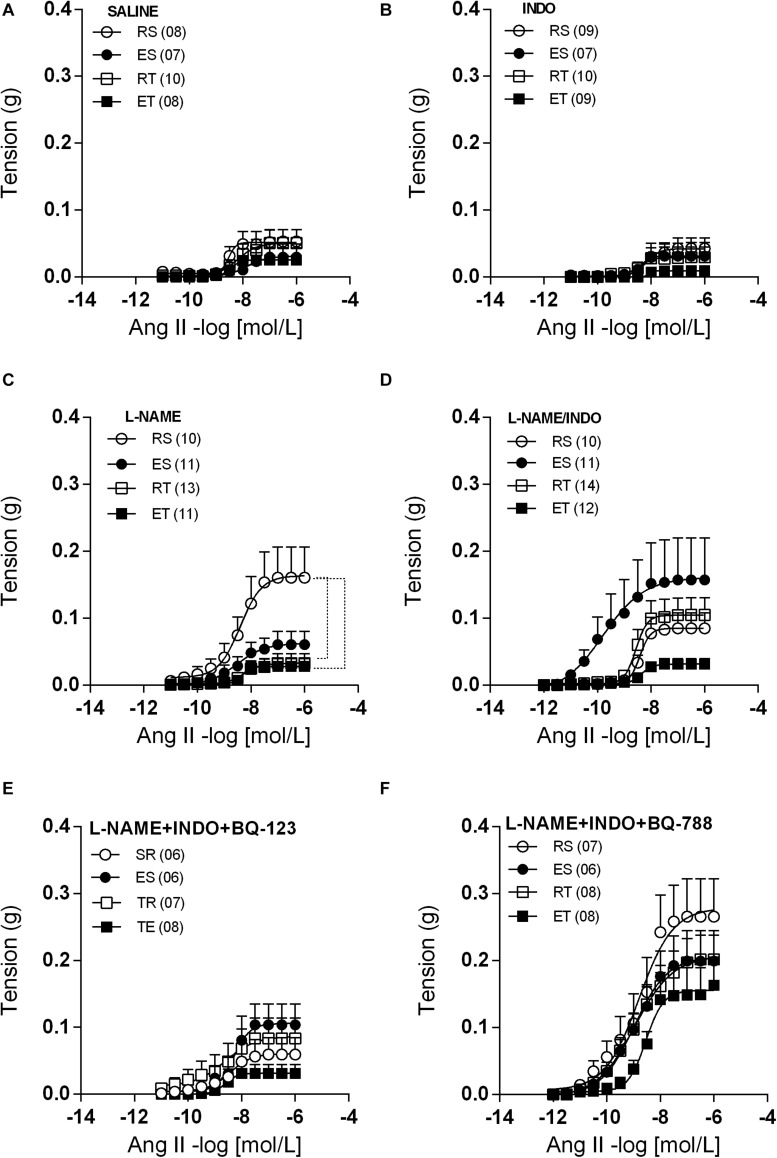
Ang II concentration-response curves determined in circular preparations of the femoral veins taken from resting-sedentary (RS), exercised-sedentary (ES), resting-trained (RT), and exercised-trained (ET) 2K1C rats, treated with saline **(A)**, 10^– 5^ mol/L indomethacin (INDO; **B**), 10^–4^ mol/L L-NAME **(C)**, 10^–4^ mol/L L-NAME plus 10^–5^ mol/L INDO **(D)**, 10^–4^ mol/L L-NAME/10^–5^ mol/L INDO plus 10^–6^ M BQ-123 **(E)** or 10^–6^ M BQ-788 **(F)**. Points represent the mean ± SEM, and the number of independent determinations is in parentheses. Dotted lines indicate groups that presented differences in terms of Rmax and AUC (see Tables 1, 2).

Preparations of femoral veins taken from 2K1C animals presented very small contractile responses to Ang II (Figure 2A). These Ang II responses were equally modest in preparations pre-treated with indomethacin (Figure 2B). On the other hand, L-NAME pretreatment increased the Ang II responses about three times only in femoral veins taken from RS animals (Figure 2C). Thus, the Ang II Rmax and AUC values obtained in these animals were significantly higher in comparison to those obtained in trained animals, either kept at rest or exposed to a single bout of exercise (Tables 1, 2). The co-incubation with L-NAME and indomethacin increased the responses of Ang II in femoral veins taken from both ES and RT animals, but not in veins taken from ET animals. Actually, in this condition, sedentary and trained animals presented similar Ang II responses at rest. However, sedentary animals exhibited higher Ang II responses when submitted to a single bout of exercise while the opposite occurred with trained animals. Notably, the Ang II responses were widely dispersed within the ES group. Thus, no difference of R_max_ was observed between groups in presence of both L-NAME and indomethacin (Figure 2D and Table 1). It should be noted that the AUC also did not differ between these groups (Table 2).

**TABLE 1:** Values of Ang II maximum response (Rmax).

Treatments	Groups			
	Resting-sedentary	Exercised-sedentary	Resting-trained	Exercised-trained
Saline	0.06 (0.00–0.10) [08]	0.01 (0.01–0.04) [07]	0.02 (0.00–0.11] [10]	0.00 (0.00–0.04) [08]
INDO	0.03 (0.00–0.09) [09]	0.02 (0.00–0.05) [07]	0.00 (0.00–0.03) [10]	0.00 (0.00–0.01) [09]
L-NAME	0.14 (0.05–0.25) [10]	0.04 (0.01–0.11) [11]	0.01* (0.00–0.04) [13]	0.00* (0.00–0.05) [11]
L-NAME+INDO	0.08 (0.02–0.12) [10]	0.05 (0.01–0.23) [11]	0.06 (0.05–0.16) [14]	0.02 (0.01–0.06) [12]
L-NAME+INDO+BQ-123	0.05 (0.02–0.09) [06]	0.10 (0.04–0.16) [06]	0.05 (0.02–0.18) [07]	0.01 (0.00–0.06) [08]
L-NAME+INDO+BQ-788	0.27 (0.09–0.43) (07)	0.20 (0.13–0.26) [06]	0.21 (0.12–0.30) [08]	0.16 (0.05–0.23) [08]
PD 123,319	0.02 (0.00–0.05) (08)	0.16* (0.04–0.23) [07]	0.03 (0.00–0.11) [09]	0.00 (0.00–0.07) [06]
Apocynin	0.04 (0.00–0.08) [05]	0.00 (0.00–0.03) [08]	0.00 (0.00–0.02) [06]	0.00 (0.00–0.04) [05]
TIRON	0.01 (0.00–0.10) [05]	0.00 (0.00–0.01) [09]	0.00 (0.00–0.04) [06]	0.00 (0.00–0.00) [05]

[n] Sample size; values expressed by median and interquartile interquartile (25th – 75th), the comparison between the groups was performed by the Kruskal–Wallis test. *indicates significant difference (*p* < 0.05) in relation to the RS group.

**TABLE 2:** Values of Ang II area under curve (AUC).

Treatments	Groups			
	Resting- sedentary	Exercised-sedentary	Resting-trained	Exercised-trained
Saline	0.12 (0.00–0.28) [08]	0.03 (0.00–0.13) [07]	0.06 (0.00–0.29) [10]	0.00 (0.00–0.08) [08]
INDO	0.07 (0.00–0.18) [09]	0.04 (0.00–012) [07]	0.00 (0.00–0.07) [10]	0.00 (0.00–0.03) [09]
L-NAME	0.35 (0.15–0.51) [10]	0.09 (0.01–0.28) [11]	0.02* (0.00–0.10) [13]	0.00* (0.00–0.18) [11]
L-NAME+INDO	0.19 (0.06–0.30) [10]	0.20 (0.02–0.89) [11]	0.19 (0.11–0.39) [14]	0.08 (0.01–0.13) [12]
L-NAME+INDO+BQ-123	0.13 (0.04–0.24) [06]	0.22 (0.09–0.49) [06]	0.12 (0.05–0.46) [07]	0.03 (0.01–0.17) [08]
L-NAME+INDO+BQ-788	0.79 (0.20–1.35) (07)	0.61 (0.47–0.77) [06]	0.73 (0.33–0.78) [08]	0.39 (0.13–0.57) [08]
PD 123,319	0.03 (0.00–0.11) (08)	0.34 (0.04–0.64) [07]	0.07 (0.00–0.27) [09]	0.00 (0.00–0.18) [06]
Apocynin	0.09 (0.00–0.23) [05]	0.01 (0.00–0.09) [08]	0.00 (0.00–0.07) [06]	0.00 (0.00–0.01) [05]
TIRON	0.01 (0.00–0.34) [05]	0.00 (0.00–0.02) [09]	0.00 (0.00–0.13) [06]	0.00 (0.00–0.00) [05]

[n] Sample size; values expressed by median and interquartile interquartile (25th – 75th), the comparison between the groups was performed by the Kruskal–Wallis test. *indicates significant difference (*p* < 0.05) in relation to the RS group.

**FIGURE 3:**
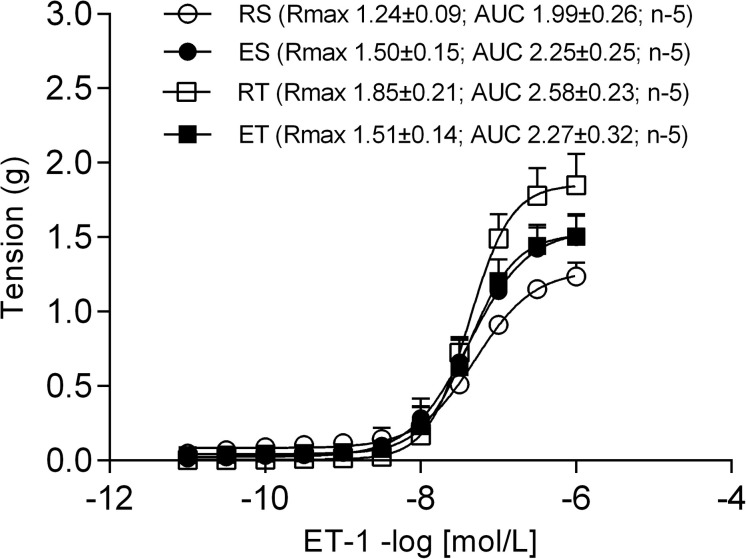
Endothelin-1 (ET-1) concentration-response curves determined in circular preparations of the femoral veins taken from resting-sedentary (RS), exercised-sedentary (ES), resting-trained (RT), and exercised-trained (ET) 2K1C rats, treated with both 10^–4^ mol/L L-NAME and 10^–5^ mol/L indomethacin (INDO). Points represent the mean ± SEM, and the number of independent determinations is in parentheses.

When BQ-123 was added to incubation, together with L-NAME and indomethacin, the Ang II responses obtained in all groups came closer to those obtained in the ET animals. In this manner, the exercise-induced changes of Ang II responses, that occurred in the presence of only L-NAME and indomethacin (Figure 2D and Tables 1, 2), were no longer observed (Figure 2E and Tables 1, 2). In contrast, when the BQ-788 was added in the incubation medium, together with L-NAME and indomethacin, the values of Ang II R_max_ were elevated in all groups of animals (Figure 2F). Thus, the exercise-induced changes of Ang II responses, previously observed in the presence of only L-NAME and indomethacin (Figure 2D and Tables 1, 2), were no longer detectable. In addition, neither the exercise training nor the exposition of sedentary or trained animals to single bout of exercise resulted in significant changes of ET-1 responses in preparations pre-treated with both L-NAME and indomethacin. In this manner, no differences of ET-1 R_max_ or pD_2_ were observed among groups (Figure 3).

**FIGURE 4:**
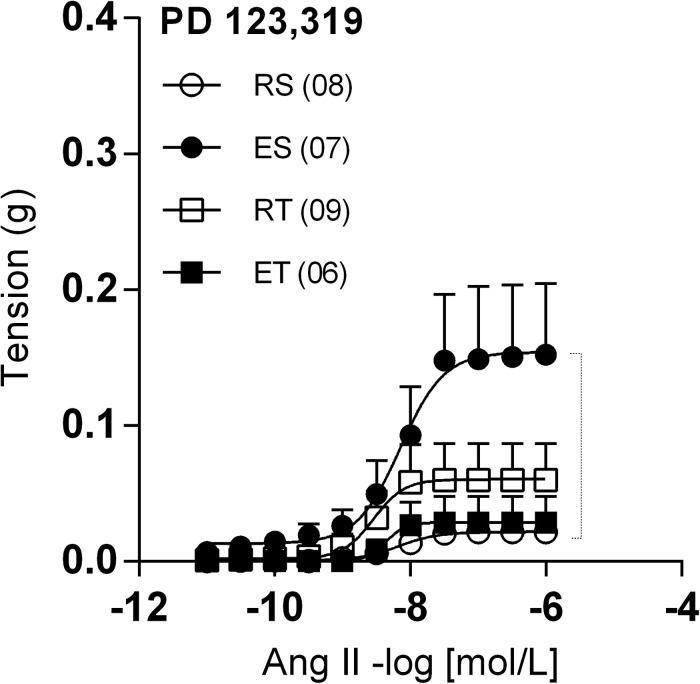
Ang II concentration-response curves determined in circular preparations of the femoral veins taken from resting-sedentary (RS), exercised-sedentary (ES), resting-trained (RT), and exercised-trained (ET) 2K1C rats, treated with 10^–6^ mol/L PD 123,319. Points represent the mean ± SEM, and the number of independent determinations is in parentheses. Dotted lines indicate groups that presented differences in terms of Rmax (see Table 1).

**FIGURE 5:**
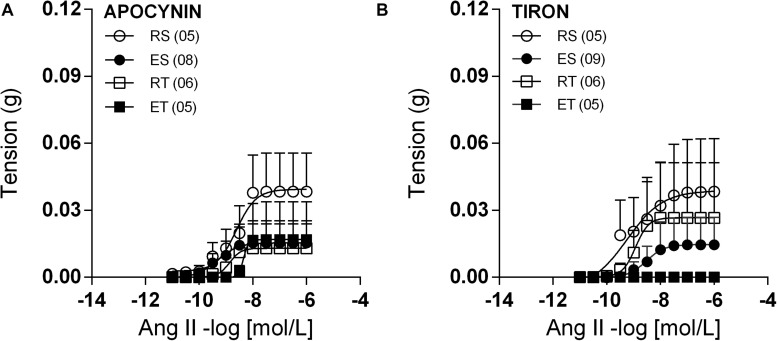
Ang II concentration-response curves determined in circular preparations of the femoral veins taken from resting-sedentary (RS), exercised-sedentary (ES), resting-trained (RT), and exercised-trained (ET) 2K1C rats, treated with 10^–4^ mol/L apocynin **(A)** or 10^–4^ mol/L tiron **(B)**. Points represent the mean ± SEM, and the number of independent determinations is in parentheses.

In the presence of PD123,319, femoral veins taken from ES animals exhibited higher responses to Ang II in comparison to the other groups (Figure 4). In line with these modifications of response, Ang II Rmax values were significantly higher in preparations of ES animals when compared to those of RS animals (Table 1). Further, the Ang II responses remained very small in the presence of apocynin (Figure 5A) or tiron (Figure 5B). Moreover, in the presence of these inhibitors, there was no exercise-induced modification of Ang II response among the groups (Figures 5A,B and Tables 1, 2).

### COX-1 and COX-2 Quantification

**FIGURE 6:**
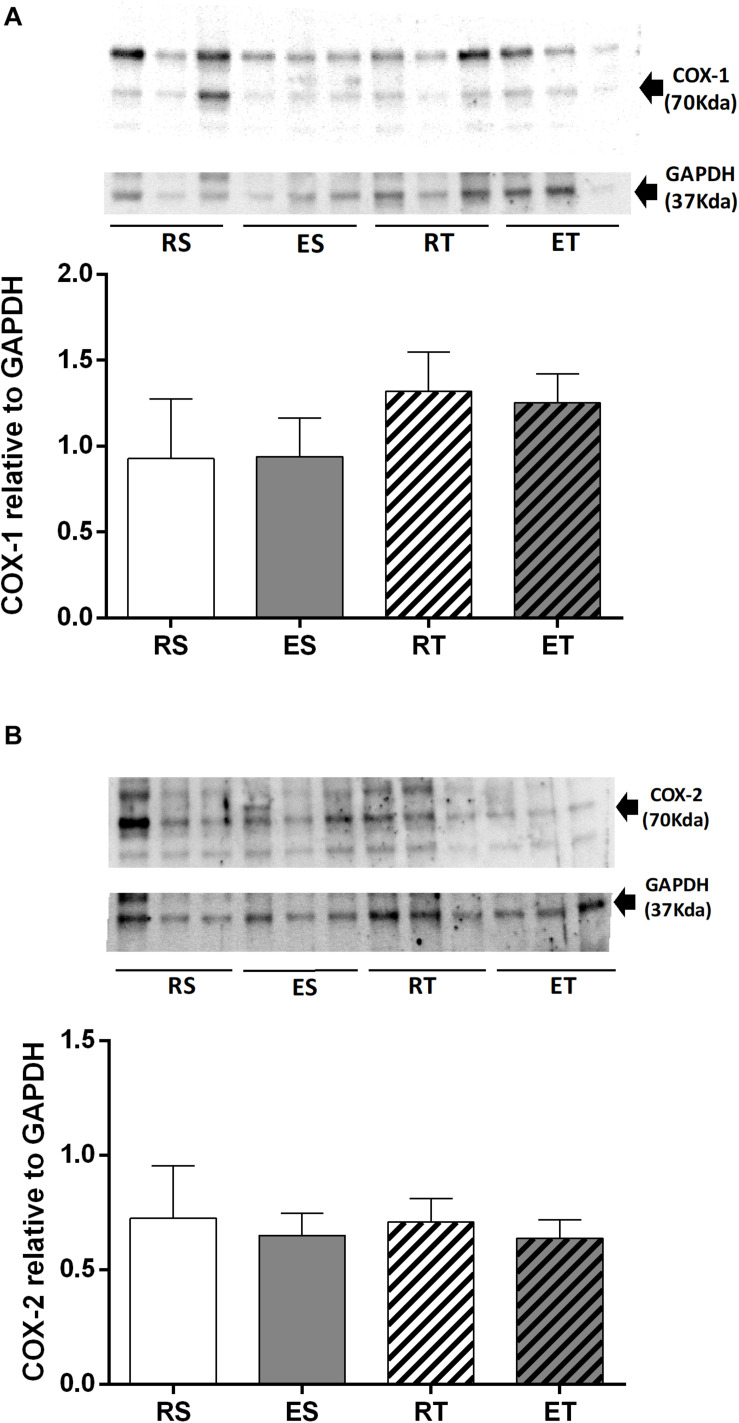
Western blotting and semiquantitative analysis of COX-1 **(A)** COX-2 **(B)**, estimated by the density of proteins bands (normalized relative to GAPDH), determined in femoral veins taken from resting-sedentary (RS), exercised-sedentary (ES), resting-trained (RT), and exercised-trained (ET) 2K1C rats. Bars indicate mean ± S.E.M of 4–5 samples.

The presence of COX-1 in femoral veins of 2K1C animals was not significantly different among the groups (Figure 6A). Likewise, there were also no differences in the levels of COX-2 among groups (Figure 6B).

## Discussion

In normotensive animals, mechanisms related to vasodilator prostanoids and/or a vasodilator mechanism mediated by ET_B_ appear to act as backup to NO to keep the Ang II responses in femoral veins markedly reduced during exercise (Chies et al., 2013). This local modulation of Ang II responses may be even more necessary in 2K1C animals, which is a hypertension model that has activation of the renin-angiotensin-aldosterone system due to increased renin secretion by the clipped kidney leading to increases in circulating renin and ANG II levels (Martinez-Maldonado, 1991; Ceron et al., 2010). Previous studies have reported high plasma Ang II levels in 2K1C rats up to 4 weeks post clipping (Martinez-Maldonado, 1991; Nakada et al., 1996). However, Guan et al. (1992) observed an increment in Ang II plasma levels at 7 days, with a return to normal plasma levels around the 25th day after clipping. In the present study, there was no difference in plasma Ang II levels between 2K1C and 2K animals when they are not submitted to exercise. Moreover, neither the acute exercise nor exercise training changed the plasma Ang II levels in 2K1C animals. Although there were no modifications of plasma Ang II levels, the hypertension model was effective since all 2K1C animals had a significant elevation of systolic blood pressure 4 weeks after clipping. These data, however, do not negate the modulatory role of the Ang II response in the femoral veins during exercise, even if the concentration of this peptide is normal in plasma. Actually, during the fourth week of the 2K1C hypertension, when the plasma levels of Ang II trend to return to normal, the tissue levels of Ang II are still elevated (Guan et al., 1992). On the other hand, we also cannot rule out a faster cleavage of Ang II by the angiotensin-2 converting enzyme (ACE-2) into these femoral veins, converting it to Angiotensin 1-7 (Fernandes et al., 2011).

As observed in normotensive animals (Chies et al., 2013), different local mechanisms appear to act coordinately to mitigate the Ang II responses in femoral veins taken from 2K1C animals. In preparations from this group, Ang II responses were unchanged by exercise. However, during NO synthesis inhibition by L-NAME, the Ang II Rmax and AUC in femoral veins were increased, but only in animals that were not exposed to any exercise. In the presence of both indomethacin and L-NAME, the responses of Ang II were higher in different groups, with the exception of the ET group. These modifications of Ang II responses, however, were not followed by statistically significant differences of Rmax or AUC because the data were widely dispersed within the ES group. These data suggest that, like in normotensive animals (Chies et al., 2013), both acute exercise and exercise training mobilizes vasodilator prostanoids to act as backup to NO (Farouque and Meredith, 2003) in order to keep under control the Ang II responses in femoral veins of 2K1C animals. In ET rats, the femoral vein responses to Ang II remained reduced even in the presence of both indomethacin and L-NAME, which suggests the participation of additional mechanisms, not related to NO or prostanoids.

Exercise can increase the shear stress on the endothelium of veins (Cheng et al., 2003). In endothelial cell cultures, it has been shown that shear stress induces expression of several enzymes, including endothelial nitric oxide synthases (eNOS) and COX-2 (Uematsu et al., 1995; Topper et al., 1996; Xiao et al., 1997; Inoue et al., 2002). Thus, an increased amount of COX-1 and COX-2 supports an enhanced participation of vasodilator prostanoids suppressing the Ang II responses in femoral veins taken from animals submitted to exercise. In the present study, however, there were no statistically significant differences in either the COX-1 or COX-2 expression among the groups. These data suggest that the exercise-induced augmentation in the influence of vasodilator prostanoids on the Ang II responses in femoral veins may be due to an increment in COX(s) activity or to a higher smooth muscle sensitivity to vasodilator prostanoids.

The data obtained in presence of both L-NAME and indomethacin, although dispersed, suggest that the Ang II responses in femoral veins are enhanced whenever sedentary animals are exposed to acute exercise. This acute exercise-evoked potentiation of Ang II responses, however, can only be observed if the synthesis of NO and vasodilator prostanoids are blocked. It was reported that Ang II can release ET-1 from endothelial cells of rat aorta (Oriji, 1999) and bovine carotid artery (Emori et al., 1989) as well as from cultured rat vascular smooth muscle cells (VSMC; Hanehira et al., 1997) by a mechanism that involves AT1 stimulation and activation of kinase C (PKC) protein. Thus, ET-1 production may be increased by exercise, but its vascular effects can also be attenuated by other endothelial mediators in these conditions (Maeda et al., 1997; Rapoport and Merkus, 2017). In this manner, it is reasonable to infer that in femoral veins of 2K1C animals submitted to acute exercise, Ang II releases ET-1 inasmuch as it stimulates AT_1_ and, as a consequence, activates PKC (Karnik et al., 2015). This released ET-1, in turn, may augment the Ang II responses in these preparations by acting on ET_A_. Such potentiation of Ang II responses could remain suppressed by NO and vasodilator prostanoids. This hypothesis is supported by findings that the increased Ang II response observed in preparations of ES animals was no longer observed when BQ-123 was added to the incubation.

When BQ-788 was added in the incubation medium, together with L-NAME and indomethacin, the values of Ang II Rmax were elevated in all groups. This augmented Ang II response caused by BQ-788 was particularly evident in preparations taken from ET animals. Preparations taken from ET animals exhibited Ang II responses greater than those observed in the other groups. This suggests that, in femoral veins of ET 2K1C animals, the Ang II has its contractile effects attenuated by relaxing mechanisms, beyond those related to NO or vasodilator prostanoids, but which are activated through ET_B_ stimulation. Supporting this inference, several studies have suggested that activation of ET_B_ releases endothelium-derived relaxing substances that attenuate the vasoconstriction mediated by ET_A_ in the vascular smooth muscle (Just et al., 2005; Maguire and Davenport, 2015). Moreover, Tirapelli et al. (2005) proposed that the ET_B_-mediated vasodilation in carotid artery involves not only NO and vasodilator prostanoids, but also activation of voltage-dependent K+ channels. Thus, it is likely that Ang II responses in femoral veins of 2K1C animals are also modulated by hyperpolarizing mechanisms activated as a consequence of ET_B_ stimulation. Unfortunately, it was not possible to identify the mediator(s), released by ET_B_ stimulation in these preparations.

The data obtained in presence of both BQ-123 and BQ-788 suggest that exercise mobilizes mechanisms through ET_A_ and ET_B_ receptors to regulate the Ang II responses in femoral veins taken from 2K1C animals. Notably, the participation of these receptors in the modulation of Ang II responses has similarities with that observed in femoral veins of normotensive animals submitted to exercise (Chies et al., 2013). This suggests that 2K1C hypertension does not substantially modify the functioning of local mechanisms that regulate the responses of these veins to Ang II.

Because of the possibility that Ang II responses in femoral veins may be influenced by ET-1, we decided to verify contractile effects of exogenous ET-1 in these preparations. This challenge was performed in the presence of both L-NAME and indomethacin in order to avoid NO and/or prostanoids masking the effects of exercise on the ET-1 responses. In these experiments, however, the exposure to exercise did not modify significantly the ET-1 responses in both sedentary and trained animals. These data suggest that the vascular responsiveness to ET-1 is not modified by exercise. However, it is noteworthy that the challenge with ET-1 was made in the absence of Ang II, which does not definitely exclude modifications of the ET-1 responses.

Interestingly, the presence of PD123,319 caused greater Ang II R_max_ in ES animals, in comparison to the RS animals. In parallel, the opposite occurred in trained animals, which exhibited reduced Ang II R_max_ immediately after a session of exercise. This pattern of response is similar to that observed in presence of both L-NAME and indomethacin, suggesting that the Ang II releases NO and prostanoids in femoral veins of animals exposed to the acute exercise through AT_2_ stimulation. It is worth mentioning, however, that elevations of Ang II responses in presence of PD123,319 were not observed in femoral veins of normotensive animals submitted to acute exercise (unpublished own data). This indicates that, in 2K1C animals, AT2-mediated mechanisms become essential to modulate Ang II responses in acute exercise situations.

The Ang II responses appear to be under constant modulation in the studied femoral veins, despite the high level of oxidative stress in 2K1C animals (Ceron et al., 2010; Campagnaro et al., 2013; Xue et al., 2015; Kumral et al., 2016). These data were unexpected because the NO, that appears to be essential in the modulation of Ang II responses in femoral veins of animals not exposed to exercise, may have its bioavailability reduced by the oxidative stress (Santilli et al., 2015; Korsager Larsen and Matchkov, 2016). In addition, Ang II responses were not affected either by tiron, a free radical scavenger, or apocynin, a NAD(P)H oxidase inhibitor. These data reinforce the notion that the 2K1C-related oxidative stress is not sufficient to impair significantly the modulation exerted by NO upon the Ang II responses in femoral veins.

**FIGURE 7:**
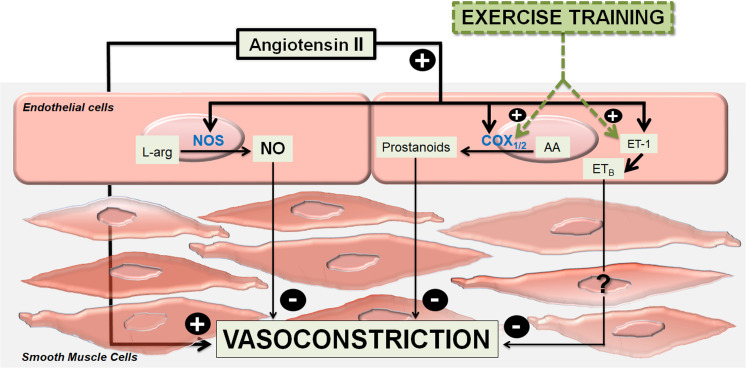
Summary graph. Modulations of angiotensin II-induced vasoconstriction in femoral veins of exercised 2-kidney-1-clip hypertensive rats (2K1C). The main vasodilator endothelial mechanism, which modulates the angiotensin II-induced vasoconstrictor effects is nitric oxide (NO) formed by the enzymes nitric oxide synthases (NOS) from L-arginine (L-Arg). Under training, vasodilator prostanoids formed by cyclooxygenase enzymes (COX1 / 2) from arachidonic acid (AA), as well as vasodilator mediators not yet identified that are released via the angiotensin II – Endothelin-1 (ET-1) – ET_B_ receptors (ET_B_), are mobilized to act cooperatively to nitric oxide.

In conclusion, the present study indicates that, in a similar manner as occurs in normotensive animals, a complex interaction of local modulating mechanisms keep the Ang II responses subdued in femoral veins of the 2K1C animals. NO-related mechanisms mitigate the Ang II responses in femoral veins of animals kept at rest. On the other hand, vasodilator prostanoids as well as other relaxing mechanisms related to ET-1 are mobilized by exercise to cooperate with NO in order to control Ang II responses in femoral veins of these animals (Figure 7).

## Data Availability Statement

The original contributions presented in the study are included in the article/Supplementary Material, further inquiries can be directed to the corresponding author/s.

## Ethics Statement

The animal study was reviewed and approved by Research Ethics Committee of the Marília Medical School (Protocol no. 300/14).

## Author Contributions

AC, MS, DC, and LN contributed to design the experiments in the study. RD, PO, TM, RM, and CR took part in the experiments. AB and AS contributed to analyze the data and writing the manuscript. DC and LN helped to perform the analysis with constructive discussions as well as reviewing the writing. All authors contributed to the article and approved the submitted version.

## Conflict of Interest

The authors declare that the research was conducted in the absence of any commercial or financial relationships that could be construed as a potential conflict of interest.
